# Effects of Inhibitors Generated by Dilute Phosphoric Acid Plus Steam-Exploded Poplar on *Saccharomyces cerevisiae* Growth

**DOI:** 10.3390/microorganisms10071456

**Published:** 2022-07-19

**Authors:** Yanan Wang, Peng Zhan, Lishu Shao, Lin Zhang, Yan Qing

**Affiliations:** 1 School of Materials Science and Engineering, Central South University of Forestry and Technology, Changsha 410004, China; wangyanan3202@163.com (Y.W.); lishushao@csuft.edu.cn (L.S.); zhlin-331@163.com (L.Z.); qingyan0429@163.com (Y.Q.); 2 Hunan International Joint Laboratory of Woody Biomass Conversion, Central South University of Forestry and Technology, Changsha 410004, China

**Keywords:** inhibitors, *Saccharomyces cerevisiae*, growth, steam explosion, poplar

## Abstract

The pretreatment of lignocellulosic biomass is important for efficient bioethanol conversion, but causes undesirable by-products that inhibit microbial growth, conversely affecting the bioconversion efficiency. In this study, the main inhibitors derived from dilute phosphoric acid plus steam-exploded poplar wood were identified as 0.22 g/L furfural, 3.63 g/L acetic acid, 0.08 g/L syringaldehyde, etc., indicating the green nature and low toxicity of the pretreatment process. The effects of the three typical inhibitors (furfural, acetic acid, and syringaldehyde) on *Saccharomyces cerevisiae* 1517RM growth were analyzed and shown to prolong the lag phase of microbial growth to different degrees. In all the inhibitor groups, the ergosterol secretion was boosted, indicating low cell membrane fluidity and robustness of the strain to an adverse environment. The cell electronegativity and morphology of *S. cerevisiae* 1517RM also changed under different growth conditions, which was helpful for monitoring the physicochemical properties of cells. Furfural, acetic acid, and syringaldehyde had a synergistic effect on each other, providing an important reference to improving the subsequent ethanol fermentation process.

## 1. Introduction

Bioethanol conversion from abundant and renewable lignocellulosic biomass holds great promise as alternatives to fossil fuel for mitigating global climate change [1]. Due to its strong bio-recalcitrance, suitable pretreatments are required to deconstruct the lignocellulosic biomass main components (cellulose, hemicellulose and lignin) and release fermentable carbohydrates for bioethanol production. Up to now, several pretreatment techniques have been developed, including physical, chemical, physicochemical, and biological processes, along with the combinations of these strategies [2]. Among them, steam explosion has advantages such as being green, efficient, and low cost, so it is recognized as a promising pretreatment technique [3]. In recent years, dilute phosphoric acid plus steam explosion (PASE) has a research direction due to its low sugar loss and the toxins generated than the traditional steam explosion processes [4,5]. Furthermore, phosphoric acid not only serves as a nutrient element for subsequent fermentation strains, but the phosphorus can also be recovered for fertilizer production [6]. 

Generally, lignocellulosic biomass pretreatment processes generate variable by-products that adversely affect bioethanol production strains such as *Saccharomyces cerevisiae* [7]. Depending on the nature of the feedstock and pretreatment technique, the types of inhibitors generated are classified into three categories: furan derivatives, carboxylic acids and phenolic compounds. Furan derivatives (i.e., furfural, hydroxymethylfurfural (5-HMF)) are formed by the dehydration of pentose and hexose, especially in the acidic and high-temperature pretreatment process [8]. Furfural is recognized as one of the most important fermentation inhibitors for many strains. Carboxylic acids such as acetic acid, formic acid, lactic acid, and levulinic acid can be generated from different fractions of lignocellulosic biomass [9]. Among them, acetic acid, which is derived from the deacetylation of hemicellulose and lignin under conditions such as high temperature and levulinic acid decomposition, is the most common carboxylic acid in the biomass pretreatment hydrolysate with concentrations of 1.0–10.0 g/L [10]. Moreover, levulinic acid may be present in the lignocellulosic hydrolysate in high concentrations, but is strongly dependent on the pretreatment conditions (such as acid catalyzed condition). Phenolic compounds (i.e., syringaldehyde, 4-hydroxybenzaldeyde, catechol, benzyl alcohol, cinnamaldehyde, and benzoic acid) are produced by lignin breakdown and carbohydrate degradation during the acidic pretretment process [11]. Generally, titers of phenolic compounds are relatively low in the lignocellulosic hydrolysate.

The inhibitor effects of the by-products generated in the pretreatment process of lignocellulosic biomass are suppressing the cell growth rate, limiting the sugar consumption, and lowering the ethanol yield when fermenting glucose using *S. cerevisiae* as biocatalyst [12,13]. Furthermore, the toxicity of furfural to *S. cerevisiae* is caused by its involvement in aldehyde reduction, pentose phosphate pathway, DNA/RNA transcriptional and translational control, stress response, etc. [13,14,15,16] The toxic effect of weak acids such as acetic acid is involved in carbohydrate and amino acid metabolism, signal transduction, material transport, etc. [17,18,19] The inhibition of phenolic compounds is caused by their hydrophobic properties, which result in partial and even overall destruction of cell membranes, weakening microbe’natural barriers against outside influence [8]. Moreover, the phenolic compounds have been shown to be toxic to both microorganism and cellulase. Generally, the effects of inhibitors on fermentation process varied depending on the type and dose of inhibitors and microbes [20,21]. Furthermore, synergistic or antagonistic effects also emerge due to the inhibitor cocktails generated [22]. To eliminate the negative effects of these inhibitors on microorganism growth and fermentation efficiency, a deeper understanding of inhibitory mechanisms and interactions is required. As a ubiquitous component of cellular membranes in yeast, ergosterol is responsible for many functions of biofilm, such as stabilizing the cell membrane structure by binding with phospholipids and regulating the selective permeability and fluidity of the cell membrane [23]. Under inhibitor stress, ergosterol can be overexpressed by *S. cerevisiae* to improve cell membrane stability for adapting to an adverse environment and counteracting the exoteric stress of inhibitors [24,25]. 

The aims of this work are to assess the performance of the PASE process and study the effects of inhibitors derived from poplar pretreated by the PASE process on *S. cerevisiae* growth. First, the main inhibitors produced in the process of PASE pretreating poplar were identified by gas chromatography–mass spectrometry (GC-MS) and high-performance liquid chromatography (HPLC) system. Then, the effects of individual inhibitors and a combination of three typical ones (furfural, syringaldehyde, and acetic acid) identified from the PASE process on *S. cerevisiae* 1517RM growth were evaluated. Moreover, the ergosterol content, zeta potential and cell morphology of different experimental groups were characterized, which represent the *S. cerevisiae* 1517RM growth states and serve as useful insight for the optimization of ethanol production.

## 2. Materials and Methods

### 2.1. Raw Materials

European black poplar (*Populus nigra* L.) sawdust (diameter: 1–2 mm, length: 2–15 mm) was pretreated according to our previous study [26], with the PASE parameters as follows: substrate presoaked in 2% (*w*/*w*) phosphoric acid at a solid–liquid ratio of 1:2.5 for 1 h, 2 MPa (215 °C) explosion pressure, and 180 s residence time. The exploded spent liquor was separated by a centrifuge at 1000× *g* r/min, and the spent liquor was collected for further analysis. 

### 2.2. Microorganism and Cultivation

*S. cerevisiae* 1517RM is a mutant of parent strain *S. cerevisiae* CICC 1517 purchased from the China Center of Industrial Culture Collection (CICC, Beijing, China), and can ferment the sugars derived from lignocellulosic biomass to ethanol. It was stored in our laboratory at −80 °C in glycerin tubes. First, *S. cerevisiae* 1517RM was incubated on a yeast peptone dextrose agar medium (10 g/L yeast extract, 20 g/L peptone, 20 g/L glucose, and 20 g/L agar) at 30 °C for 24 h. Subsequently, the yeast cells were transferred to a 250 mL flask containing 100 mL of yeast peptone dextrose medium at 30 °C and 150 revolutions per minute for 24 h, then used as inoculums. 

The cultivation experiments were performed in 100 mL Erlenmeyer flasks containing 50 mL of medium (40 g/L glucose, 5 g/L (NH_4_)_2_SO_4_, 2.0 g/L KH_2_PO_4_, 0.4 g/L CaCl_2_, 0.4 g/L MgSO_4_∙7H_2_O dissolved in 0.05 M citric acid buffer at pH 4.8) and 5.0% (*w*/*w*) (1.0 g/L cell dry weight) yeast inoculation, maintained at an initial pH of 5.0 and not adjusted anymore. The flasks were incubated at 30 °C and 150 r/min for 72 h.

The individual and combined effects of inhibitors on the growth of *S. cerevisiae* 1517RM were evaluated by adding different concentrations of single inhibitors or a combination of three (furfural, acetic acid, and syringaldehyde; the concentrations are given in the Results and Discussion section of this work) dissolved in dimethyl sulfoxide into a sterile fermentation medium. Equal amounts of dimethyl sulfoxide were added to the blank group. The chemical agents except furfural, acetic acid, and syringaldehyde were analytical grade and purchased from Sinopharm, Chemical Reagent Co., Ltd. (Shanghai, China). Furfural, acetic acid, and syringaldehyde were guaranteed reagents, purchased from Shanghai Aladdin Biochemical Technology Co., Ltd. (Shanghai, China).

### 2.3. PASE Spent Liquor Composition

The inhibitors of PASE spent liquor were qualitatively analyzed with a GC-MS system (America Agilent Technologies, Palo Alto, CA, USA, 7080B GC system and 5977A MSD). The spent liquor was first extracted by diethyl ether. Then the organic fraction was dehydrated using magnesium sulfate anhydrous for subsequent detection. The injector and transfer line temperatures were set to 230 °C. The temperature program was as follows: 2 min at 80 °C, then ramp up to 330 °C with a heating rate of 15 °C/min. Helium was used as the carrier gas, with a column flow rate of 1 mL/min.

Furfural and 5-HMF were separated on an HPLC system equipped with an AlltimaTM C18 column (Berkshire, UK) and an ultraviolet detector at 285 nm. The syringaldehyde and vanillin contents were determined using the same HPLC system as for furfural, with a detector wavelength of 280 nm. The chemical agents furfural, 5-HMF, acetic acid, and syringaldehyde were used without further purification.

Glucose, xylose, and acetic acid were detected using a refractive index detector and an Aminex HPX-87H column (300 mm × 7.8 mm × 5 µm, Bio-Rad, Hercules, CA, USA) according to the American national renewable energy laboratory (NREL) standard method [27]. 

### 2.4. S. cerevisiae 1517RM Biomass Measurement

The biomass of *S. cerevisiae* 1517RM was expressed as the optical density of 600 nm (OD_600_) using a Shimadzu UV-2450 spectrophotometry (Kyoto, Japan). After fermentation, the cells were harvested using a centrifuge at 5000× *g* r/min The precipitates considered as wet biomass were washed and diluted with deionized water to an OD_600_ of 0.2–0.8 for measuring.

### 2.5. Zeta Potential and Surface Morphology Characterization

The zeta potential of *S. cerevisiae* 1517RM cells was characterized by a Zeta potential meter (Nano ZS90 zetasizer, Malvern Instruments Corp., Worcestershire, UK). The main procedure was as follows. First, the fermentation broths of 72 h were separated by a centrifuge at 5000× *g* r/min. The precipitates considered as wet biomass were collected and washed twice with ultrapure water, then collected and frozen in a freeze dryer at −48 °C and 4 Pa for 24 h, leading to the generation of dry cell biomass. For measurement, 1 mg of dry cell biomass was diluted with ultrapure water to a constant volume of 10 mL. A certain amount of cell solution was injected into a sample cell for detection at 25 °C. The average values of three measurements were chosen as the final zeta potential of samples.

The surface morphology of *S. cerevisiae* 1517RM cells was observed using a scanning electron microscope (SEM, Hitachi SU8010, Tsukuba, Japan). The pretreatment and characterization procedure were as described by Gu et al. [28], with modifications as follows. The cells were harvested using a centrifuge at 5000× *g* r/min for 5 min, and then washed twice with 0.1 mol/L phosphate buffer solutions. The cells were mixed with 2.5% (*w*/*w*) glutaraldehyde at 4 °C for 12 h. For testing, cells were washed three times with 0.1 mol/L phosphate buffer solutions for 15 min, followed by 1% (*w*/*w*) osmic acid solution for 1h, and washed using 0.1 mol/L phosphate buffer solution for 15 min. The cells were dehydrated twice with anhydrous ethanol, followed by treatment with isoamyl acetate for 1 h. Then, the cells were dried with a critical point dryer and coated with a gold and platinum alloy before characterization.

### 2.6. Intracellular Ergosterol Determination

Intracellular sterols were extracted and calculated using the method reported by Arthington-Skaggs et al. [29] with some slight modifications as follows. First, the fermentation broths of 72 h were separated by a centrifuge at 5000× *g* r/min for 8 min, and then the precipitates considered as wet biomass were collected and washed twice with ultrapure water. The cells were collected for freeze-drying, followed by dissolved in a 10 mL tube containing 3 mL of a 25% (*w*/*w*) potassium hydroxide–ethanol solution. The tubes were treated in a water bath at 85 °C for 1 h, naturally cooled to room temperature, then mixed with 3 mL heptane and 1 mL ultrapure water. The organic phase was moved to a new tube and diluted with anhydrous ethanol to a 5:1 ratio, leaving pure ergosterol dissolved in the solutions. The absorbance of the solution was measured at 281.5 and 230 nm using a UV spectrophotometer (Shimadzu UV-2450). The concentration of ergosterol was calculated by the following formula given by Zhu et al. [30]:

Ergosterol (%) = (A_281.5_/290 − A_230_/518) × (F/B) × 100% (1)

wherein, A_281.5_ and A_230_ represent the absorbance values at 281.5 nm and 230 nm, respectively. F and B represent the dilution ratio of ergosterol in ethanol and dry cell biomass, respectively. The coefficients of 290 and 518 represent the extinction values determined for crystalline ergosterol, respectively.

## 3. Results and Discussion

### 3.1. PASE Spent Liquor Composition

As shown in Table 1, the main by-products of PASE spent liquor such as furfural, phenol, benzoic acid, 5-HMF, vanillin, vanillic acid, syringaldehyde, and coniferyl aldehyde were identified, indicating partial destruction of the substrate structure and decomposition of the lignocellulosic biomass main components (such as cellulose, hemicellulose, and lignin) into monomeric substances. Compared with the results of previous studies [31,32,33,34,35,36] (shown in Table 2), the PASE process generated fewer by-products than other pretreatment methods using poplar as a feedstock.

### 3.2. Effects of Inhibitors on S. cerevisiae 1517RM Growth

**Figure 1:**
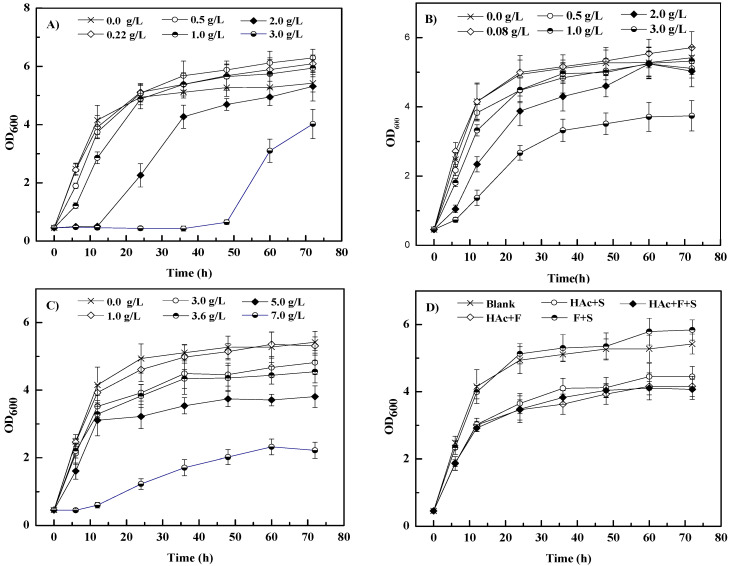
Effects of individual and cocktail of inhibitors on biomass of *S. cerevisiae* 1517RM. (i) (**A**–**D**) represents furfural, syringaldehyde, acetic acid, and inhibitor cocktail, respectively. (ii) Blank, HAc + F, HAc + S, F + S, and HAc + F + S represents 0.0 g/L inhibitor, 3.63 g/L acetic acid + 0.22 g/L furfural, 3.63 g/L acetic acid + 0.08 g/L syringaldehyde, 0.22 g/L furfural + 0.08 g/L syringaldehyde, and 3.63 g/L acetic acid + 0.22 g/L furfural + 0.08 g/L syringaldehyde, respectively. Three replicates were carried out in this work. The symbol of bars are the standard deviations.

The individual and combined effects of furfural (0–3.0 g/L), acetic acid (0–7.0 g/L), and syringaldehyde (0–3.0 g/L) on the growth of *S. cerevisiae* 1517RM are shown in Figure 1. As shown in Figure 1A, the lag phase of *S. cerevisiae* 1517RM was slightly affected with furfural less than 1.0 g/L, which was consistent with the result of a previous study carried out by da Silva et al. using *S. carlsbergensis* ATCC 6269 [37]. Along with the increase in furfural content, the lag phase was remarkably prolonged by 48 h (3.0 g/L furfural group). Interestingly, compared with the blank group (0 g/L furfural), a slight increase in biomass yield was observed over time (>24 h) in the 0.22, 0.5, and 1.0 g/L furfural groups after 72 h (Table 3). Previous studies showed that a slight increase in biomass yields using *Thermoanaerobacter pseudethanolicus* 39E [38] and *Candida tropicalis* [39] as biocatalysts was achieved at furfural ~1.0 g/L, indicating that a low concentration of furfural may play a role as a growth additive rather than an inhibitor. It was also reported that microbes can tolerate low titers of furfural and convert them to less toxic compounds such as furfuryl alcohol and furoic acid [40]. Furthermore, the reduction in furfural can partially replace glycerol formation, regenerate hydrogen receptor NAD^+^, limit carbon loss to by-products, and allow more carbon distribution of biomass synthesis [39,41]. 

As shown in Figure 1B,C, the effects of syringaldehyde and acetic acid on *S. cerevisiae* 1517RM growth were similar to those of furfural. However, the degrees of lag phase delay in syringaldehyde and acetic acid groups were lower than in the furfural group at the same concentration, especially over 1.0 g/L. The biomass yield of all groups except the 0.08 g/L syringaldehyde group was smaller than that of the blank group after 72 h, indicating some degree of inhibition on the microbial population (Table 3). The higher biomass of the 0.08 g/L syringaldehyde group than that of the blank group might be involved in the metabolic mechanism of syringaldehyde at different conditions. Moreover, the biomass of the syringaldehyde groups except the 0.08 g/L one was smaller than that of the furfural groups in the same conditions, indicating that syringaldehyde might be more toxic to *S. cerevisiae* 1517RM than furfural. Similar results have been achieved using strains such as *S. cerevisiae* [42], *Candida guilliermondi* [43], and *Cryptococcus curvatus* [44] under different experimental conditions. To date, the toxicities to different types of microbes’ cell growth stressed by acetic acid of less than 10.0 g/L have been tested intensively, producing results similar to this study [45,46,47]. The delay of the lag phase could be caused by the inhibition of nutrient uptake and the acidification of the intracellular matrix of microbe [46,47]. In addition, long-term acidification plays a key role in *S. cerevisiae* cell growth inhibition under acetic acid stress [45]. Furthermore, glucose can inhibit the transport and metabolism of acetate, leaving acetic acid as a toxic inhibitor of glucose fermentation using *S. cerevisiae* as the biocatalyst [48]. However, some yeast species such as *Yarrowia lipolytica* are able to use acetic acid as sole or mixed carbon and energy sources for growth and metabolic activities [49,50].

Up to now, many results of individual inhibition of microbes have been revealed; however, little attention has been given to the synergistic effect of inhibitors [51]. Compared with the blank group, the lag phase of all inhibitor cocktails (3.63 g/L acetic acid + 0.22 g/L furfural, 3.63 g/L acetic acid + 0.08 g/L syringaldehyde, 0.22 g/L furfural + 0.08 g/L syringaldehyde and 3.63 g/L acetic acid + 0.22 g/L furfural + 0.08 g/L syringaldehyde) was not notably influenced (Figure 1D and Table 3). The biomass of the 3.63 g/L acetic acid + 0.22 g/L furfural, 3.63 g/L acetic acid + 0.08 g/L syringaldehyde and 3.63 g/L acetic acid + 0.22 g/L furfural + 0.08 g/L syringaldehyde groups was significantly lower (*p* < 0.05) than that of the blank group when the cultivation time was over 6 h, with 76.8%, 82.1%, and 75.1% of biomass in blank group at 72 h, respectively. The biomass of the 0.22 g/L furfural + 0.08 g/L syringaldehyde group was slightly higher (*p* > 0.05) than that of the blank group when the cultivation time was over 24 h, with 107.7% of biomass in the blank group at 72 h. Overall, the biomass of the 0.22 g/L furfural + 0.08 g/L syringaldehyde group was slightly higher (*p* > 0.05) and lower than (*p* > 0.05) that of 0.08 g/L syringaldehyde and 0.22 g/L furfural group over time, with 102.3% and 95.9% of biomass in the 0.08 g/L syringaldehyde and 0.22 g/L furfural groups at 72 h, respectively. The interaction between a low concentration of furfural and syringaldehyde should be discussed in further research. Moreover, both furfural and syringaldehyde enhanced the inhibition of cell growth in an acetic acid cultivation broth, resulting in a delay of the growth profile and a decrease in biomass yield at the same inhibitor concentration. The above results indicate that furfural, acetic acid, and syringaldehyde create synergistic inhibition on the growth of *S. cerevisiae* 1517RM. Similar results were demonstrated in previous studies [21,51].

### 3.3. Effects of Inhibitors on Ergosterol Content

**Figure 2:**
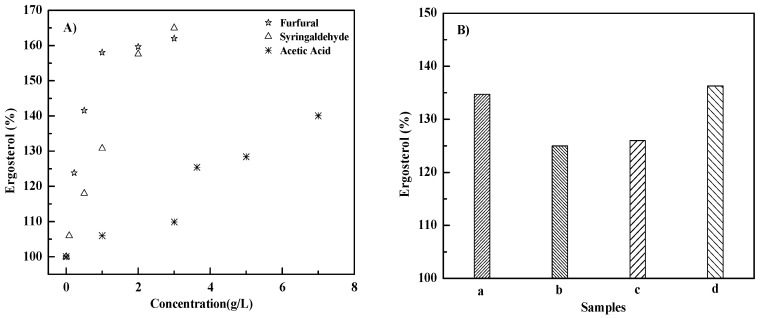
Effects of inhibitors on ergosterol at 72 h. (**A**) Individual inhibitor group; (**B**) Inhibitor cocktails. a–d represents cocktail of 3.63 g/L acetic acid + 0.22 g/L furfural, 3.63 g/L acetic acid + 0.08 g/L syringaldehyde, 0.22 g/L furfural + 0.08 g/L syringaldehyde, and 3.63 g/L acetic acid + 0.22 g/L furfural + 0.08 g/L syringaldehyde, respectively.

As shown in Figure 2, the intracellular ergosterol contents of all inhibitor groups were 6.0–65.0% higher than those of blank group (100%), and increased with the increase in the inhibitor concentration, with a maximum value of 165.0% (at 3.0 g/L syringaldehyde). Furthermore, the ergosterol contents of the furfural and syringaldehyde groups at 1.0 g/L and 3.0 g/L were higher than those of the acetic acid group at the same concentrations (Figure 2A). The reasons might be that furfural and syringaldehyde cause more damage to cell membranes than acetic acid, resulting in more ergosterol being produced by *S. cerevisiae* 1517RM to resist the adverse effects. The ergosterol in the inhibitor cocktail was 3.2–20.3% higher than that of the individual inhibitor at the same concentration, with a maximum value of 136.3% (3.63 g/L acetic acid + 0.22 g/L furfural + 0.08 g/L syringaldehyde group), which was 36.3% higher than that of the blank group (Figure 2B). The inhibitor might have a synergistic effect on *S. cerevisiae* 1517RM cells, leading to an increase in the ergosterol content. Ergosterol is the main sterol of *S. cerevisiae*, and is responsible for the permeability and fluidity of the cell membrane. As reported, a high ergosterol concentration in *S. cerevisiae* is typically associated with low membrane fluidity [52,53]. An increase in ergosterol contents of different *S. cerevisiae* strains has been observed under different inhibitor stresses such as vanillin [25] and acetic acid [19]. Summing up, the above findings of this work provide a biotechnological basis for understanding the tolerance of *S. cerevisiae* 1517RM to inhibitors identified from the PASE process.

### 3.4. Zeta Potential on of S. cerevisiae Cells

**Figure 3:**
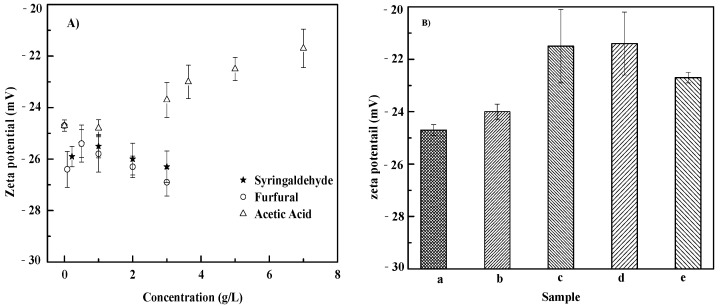
Effects of inhibitors on zeta potential at 72 h. (**A**) Individual inhibitor group; (**B**) Inhibitor cocktails. a–e represent the group of blank, cocktail of 3.63 g/L acetic acid + 0.22 g/L furfural, 3.63 g/L acetic acid + 0.08 g/L syringaldehyde, 0.22 g/L furfural + 0.08 g/L syringaldehyde, and 3.63 g/L acetic acid + 0.22 g/L furfural + 0.08 g/L syringaldehyde, respectively.

As shown in Figure 3A, the cell electronegativities of the individual furfural and syringaldehyde groups slightly increased with the growth of inhibitors (0–3.0 g/L), with zeta potentials of −24.1 mV to –26.3 mV and –26.9 mV, respectively. The cell electronegativity of the acetic acid group decreased with an increased inhibitor concentration (0–7.0 g/L), with zeta potentials from –24.1 mV to –21.7 mV. Compared with the blank group, the cell electronegativity of inhibitor cocktail decreased to an extent (Figure 3B). Moreover, the cell electronegativities of the inhibitor cocktail group were lower than those of the individual groups at the same concentration except the 3.63 g/L acetic acid + 0.22 g/L furfural group. Zeta potential is a measurement of the net distribution of electrical charge on the surface of a suspended particle, and has been applied for characterizing bacterial behavior such as changes in cell surface properties under different growth conditions [16,54]. Generally, the net charge on the surface of *S. cerevisiae* is negative, mainly caused by characteristics of the cultivation broth such as pH, ionic strength, conductivity, additive concentration, etc. Furthermore, it is reported that more negative zeta potential values are related to yeast cell wall damage [55]. The variation in the zeta potential of the cell wall reflects not only changes that occur in the cells but also changes in the broth during their growth. The slight increase in the negative zeta potential of the individual furfural and syringaldehyde groups might be caused by damage to the cell wall. The decrease in negative zeta potential of the individual acetic acid group might be a result of the increase in acidity of the broth caused by the growth of acetic acid. The results of the changes in the negative zeta potential of inhibitor cocktails were the synergistic effect of inhibitors and pH of broth, which should be the subject of more research. Moreover, further research related to the dynamics of broth pH should be performed to evaluate the effect of pH on the zeta potential of cells.

### 3.5. Surface Morphologies of S. cerevisiae Cells

**Figure 4:**
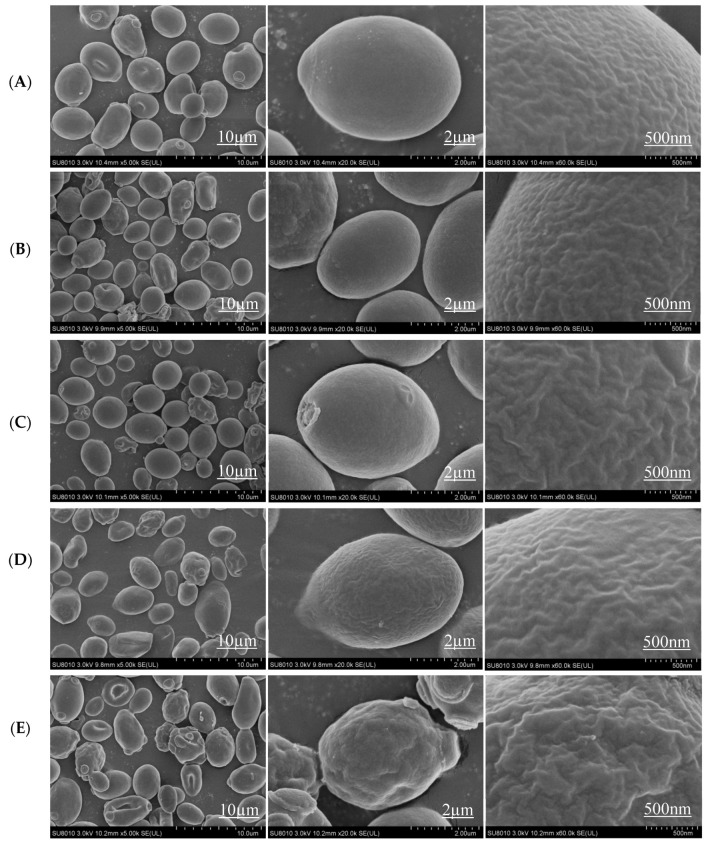
Surface morphology of *S. cerevisiae* 1517RM of different groups at 72 h. (**A**–**E**), represented blank, 3.0 g/L furfural, 7.0 g/L acetic acid, 3.0 g/L syringaldehyde, and 0.22 g/L furfural + 0.08 g/L syringaldehyde + 3.63 g/L acetic acid group, respectively.

The *S. cerevisiae* 1517RM surface morphologies of different groups after 72 h of cultivation were characterized by SEM. As shown in Figure 4A, the cells of the blank group were regular. The surface of the cells was smooth without obvious folds or cracks (Figure 4A). However, the cell density and irregularity of individual inhibitors groups increased, with certain damaged and fragmented structures (Figure 4B–D). The reasons for these changes might be a lack of energy supplies or damage to chromosomes and organelles caused by inhibitors [56]. Most of the cells were structurally intact and spherical with smooth surfaces, indicating that some damage to the cell membrane structures occurred. In addition, the cell in the acetic acid + furfural + syringaldehyde group was slightly bonded, with altered morphology and an increased degree of irregularity (Figure 4E). Comparing the cells of the blank and individual inhibitor groups, the cell surface was more uneven, rough, and very obviously crumpled, showing that combined inhibitors greatly affected the cell membrane.

## 4. Conclusions

In this study, the main inhibitors derived from dilute phosphoric acid plus steam- exploded poplar wood were identified, showing low-toxicity by-products generated during the pretreatment process. The effects of inhibitors such as furfural, acetic acid, and syringaldehyde on *S. cerevisiae* 1517RM growth were analyzed. The results showed that furfural prolonged the lag phase of cell growth, but boosted (furfural less than 1.0 g/L) biomass production. Acetic acid and syringaldehyde inhibited the strain growth, mainly by delaying cell growth and decreasing biomass. The above three inhibitors had synergistic effects on each other. Both individual inhibitors and a combination increased the ergosterol content of *S. cerevisiae*. The negative zeta potential of cells changed to some extent under different growth conditions. However, the dynamics of broth pH should be monitored in future studies. The cocktail of three inhibitors had the most significant effects on the surface morphology of *S. cerevisiae*, increasing the degree of cellular irregularity and damage and the cell surface morphology under an adverse environment, which is an important point of reference for improving the subsequent ethanol fermentation process.

## Figures and Tables

**Table 1:** GC-MS and HPLC data.

GC- MS	**Retention Time (min)**	**Compound**	**Origin**	**Retention Time (min)**	**Compound**	**Origin**
	1.878	Furfural (C_5_H_4_O_2_)	Pentose (Hemicellulose)	8.365	Vanillin (C_8_H_8_O_3_)	Guaiacyl unit (Lignin)
	3.217	Phenol (C_6_H_6_O)	ρ-Coumaryl unit (Lignin)	11.187	Vanillic acid (C_8_H_8_O_4_)	Guaiacyl unit (Lignin)
	5.695	Benzoic acid (C_7_H_6_O_2_)	ρ-Coumaryl unit (Lignin)	11.593	Syringaldehyde (C_9_H_10_O_4_)	Syringyl unit (Lignin)
	6.069	5-HMF (C_6_H_6_O_3_)	Hexose (Cellulose)	12.473	Coniferyl aldehyde (C_10_H_10_O_3_)	Guaiacyl unit (Lignin)
HPLC	Compound		Origin		Concentration (g/L)	
	Furfural (C_5_H_4_O_2_)		Pentose (Hemicellulose)		0.22	
	5-HMF (C_6_H_6_O_3_)		Hexose (Cellulose)		0.19	
	Vanillin (C_8_H_8_O_3_)		Guaiacyl unit (Lignin)		0.04	
	Syringaldehyde (C_9_H_10_O_4_)		Syringyl unit (Lignin)		0.08	
	Acetic acid (C_2_H_4_O_2_)		(Hemicellulose/Lignin)		3.63	
	Glucose (C_6_H_12_O_6_)		(Cellulose/Hemicellulose)		5.11	
	Xylose (C_5_H_10_O_5_)		(Hemicellulose)		14.88	

**Table 2:** By-products derived from poplar feedstock by different physical and chemical pretreatment techniques.

Biomass	Pretreatment Conditions	By-Products (g/L)	Reference
Yellow poplar wood (*Liriodendron tulipifera*)	Pressure cooking: less than 40 mesh size, 1:10 substrates/solvent (50% aqueous ethanol), 1% sodium hydroxide, 140–160 °C, 10 min.	Acetic acid: 4.2–4.9	[31]
Yellow poplar	Pressure cooking: 1 × 5 mm particle size, 6.0–6.6% (*w*/*w*) solids content, 220–260 °C, 70–75 min.	Furfural: 1.6–2.7; 5-HMF: 0.90–1.55; Acetic acid: 2.3–3.1	[32]
Poplar NE222 (*Populus deltoides Bartr. ex* *Marsh* × *Populus nigra* L.)	Sulfate pretreatment: 6–38 mm wood chips with 1–5 mm thicknesses, 25% (*w*/*w*) total solids loading, 2 mL/L sulfuric acid, 135 °C, 290 min.	Furfural: 0.9; Acetic acid: 16.7	[33]
Yellow poplar wood (*Liriodendron tulipifera*)	Pre-deacetylation with NaOH, Oxalic acid hydrothermal pretreatment: 20–80 mesh size, 0.16 mol/L C_2_H_2_O_4_, 150 °C, 42 min.	Furfural: 2.73–3.09; 5-HMF: 0.16–0.21; Acetic acid:not detectable	[34]
Poplar	Steam explosion: 210 °C and 4 min residence time.	Furfural: 0.49; 5-HMF: 0.08; Acetic acid: 2.1	[35]
White poplar (*Populus alba* L.)	Steam explosion: 0.7 mm particle size, pre-soaked in water for at least 1 h, 205 °C, 10 min residence time.	Furfural: 1.6; 5-HMF:0.6; Acetic acid: 4.9 (sapwood) and 6.0 (coppice)	[36]

**Table 3:** Effects of individual and cocktail of inhibitors on biomass at 72 h.

Inhibitor	Inhibitor (g/L)	Biomass * (OD)	Inhibitor	Inhibitor (g/L)	Biomass * (OD)
Blank	0.00	5.42 ± 0.32	Blank	0.00	5.42 ± 0.32
Furfural	0.22	6.09 ± 0.34	Syringaldehyde	0.08	5.71 ± 0.46
	0.50	6.29 ± 0.36		0.50	5.11 ± 0.28
	1.00	5.94 ± 0.32		1.00	5.32 ± 0.39
	2.00	5.31 ± 0.54		2.00	5.03 ± 0.45
	3.00	4.02 ± 0.53		3.00	3.74 ± 0.44
Acetic acid	1.00	5.31 ± 0.27	Inhibitor cocktail	a	4.16 ± 0.38
	3.00	4.80 ± 0.36		b	4.45 ± 0.35
	3.63	4.55 ± 0.33		c	5.84 ± 0.37
	5.00	3.81 ± 0.32		d	4.07 ± 0.34
	7.00	2.22 ± 0.24		/	/

* (i) Data shown in Table 3 are the average of three replicate experiments. (ii) a, b, c, and d represents the inhibitor cocktails of 3.63 g/L acetic acid + 0.22 g/L furfural, 3.63 g/L acetic acid + 0.08 g/L syringaldehyde, 0.22 g/L furfural + 0.08 g/L syringaldehyde, and 3.63 g/L acetic acid + 0.22 g/L furfural + 0.08 g/L syringaldehyde, respectively.

## Data Availability

Not applicable.
